# EHMTI-0172. “Calabria cephalalgic network”: innovative services and systems for the integrated clinical management of headache patients

**DOI:** 10.1186/1129-2377-15-S1-D12

**Published:** 2014-09-18

**Authors:** D Conforti, MC Groccia, B Corasaniti, R Guido, R Iannacchero

**Affiliations:** 1 DIMEG, University of Calabria, Rende, Italy; 2 Neurology Division Center for Headache Clinic, General Hospital "Pugliese-Ciaccio", Catanzaro, Italy

## Introduction

The Calabria Cephalalgic Network represents a novel healthcare delivery context which, according to a patient-centered vision, allows to effectively integrate different care settings (mainly primary and secondary care) by developing and implementing evidence based clinical workflows. On this basis, the Network is able to effectively support continuity of care, outpatient-inpatient integration, efficient use of health care resources and reduction of economic costs.

## Aims

We present the architectural organization of the network, the structure of the clinical workflows and the related technology platform providing a captivating and pro-active informative and decision making dashboard in order to effectively and efficiently support the Headache integrated care processes.

## Methods

The proposed organization of the Cephalalgic Network is based on a new integrated care program characterized by: (i) the set of relevant health care “actors” with roles and responsibilities; (ii) the services for sustaining collaborative and cooperative interactions among the end-users; (iii) the services for supporting the planning and operative management of all involved health care resources; (iv) the decision-making services for supporting integrated clinical workflows among the several healthcare settings.

## Results

The Calabria Cephalalgic Network is currently under validation by a piloting activity involving a set of healthcare actors (GPs, Hospitals, local healthcare authorities and service providers) and enrolled patients. Relevant cost-benefit indicators have been defined and collected during the validation activity.

## Conclusions

The current results confirm the effectiveness of the proposed approach, since it allow more efficient clinical service planning and management by making the best use of available hospital resources.

No conflict of interest.

